# Effect of Nanopatterning on Concentration Polarization during Nanofiltration

**DOI:** 10.3390/membranes11120961

**Published:** 2021-12-07

**Authors:** Lauren M. Ward, Barbara G. Fickling, Steven T. Weinman

**Affiliations:** Department of Chemical and Biological Engineering, The University of Alabama, Tuscaloosa, AL 35487, USA; lmward4@crimson.ua.edu (L.M.W.); bgfickling@crimson.ua.edu (B.G.F.)

**Keywords:** thin-film composite membranes, surface patterning, concentration polarization

## Abstract

Membranes used for desalination still face challenges during operation. One of these challenges is the buildup of salt ions at the membrane surface. This is known as concentration polarization, and it has a negative effect on membrane water permeance and salt rejection. In an attempt to decrease concentration polarization, a line-and-groove nanopattern was applied to a nanofiltration (NF) membrane. Aqueous sodium sulfate (Na_2_SO_4_) solutions were used to test the rejection and permeance of both pristine and patterned membranes. It was found that the nanopatterns did not reduce but increased the concentration polarization at the membrane surface. Based on these studies, different pattern shapes and sizes should be investigated to gain a fundamental understanding of the influence of pattern size and shape on concentration polarization.

## 1. Introduction

Water scarcity is a challenge that the world is facing and will continue to face as more countries become industrialized [1]. Fresh water supply is already limited around the world, and as countries become more developed, resulting in better living conditions and increasing human population, it will become a much more valued resource. Membranes are semi-permeable barriers that are used to purify water in a variety of applications. Currently, many different types of membrane processes are used to purify water. The most common pressure-driven membrane methods are microfiltration (MF), ultrafiltration (UF), nanofiltration (NF), and reverse osmosis (RO). NF and RO membranes, also called thin-film composite membranes, are comprised of a polyamide active layer, a support layer, and a non-woven fabric support, and are used to desalinate water. These membranes have different usages due to the differences in their polyamide network structures, and therefore differences in what they reject. NF membranes have characteristics similar to both UF and RO membranes [2,3]. Their pore sizes are larger than RO membranes, yet smaller than UF membranes. RO membranes are most commonly used to desalinate water and are the dominant technology for seawater desalination [4]. NF membranes are also used for desalination; however, because their polyamide layer is looser than that of RO membranes, they are often used to reject divalent ions, whereas RO membranes reject virtually all ions [5,6,7,8]. This is especially useful for water softening, where divalent ions need to be removed and monovalent ions do not. This lower rejection of monovalent ions leads to lower the transmembrane pressures required for separation, resulting in significantly lower energy costs.

Current NF and RO membranes used in water desalination face many challenges. The two most common challenges are fouling and concentration polarization. Fouling occurs when there is a blockage of the membrane pores or build-up of particles on the membrane surface. There are many different types of fouling, including scaling, organic, biofouling, and colloidal fouling [9]. There are many published studies on both chemical and physical modification methods to decrease membrane fouling and limit its effect on the membrane [10,11,12,13,14,15]. Concentration polarization is the buildup of salt ions at or just above the surface of the membrane. This is caused by the ions being rejected by the membrane while the water permeates through it. Concentration polarization can negatively affect the permeance and rejection of the membrane because of the layer of salt ions at or just above the membrane surface. The reduced water permeance is partially caused by the concentrated ion layer that prevents water molecules from getting to and permeating through the membrane. Additionally, the concentrated ion layer increases the osmotic pressure at the membrane surface, causing a decrease in the net driving force pressure, and therefore a decrease in flux. Multiple studies have been done to find ways to combat concentration polarization, including using spacers [16,17,18,19], improving membrane materials [20,21], and optimizing the module design [22,23].

Physical modification (i.e., patterning) of the membrane surface has been investigated. Some of the first patterns used to resist fouling were inspired by Nature, such as the pattern found on sharks’ skin [24,25,26,27,28,29]. Membrane surface patterning has been studied extensively to reduce fouling [9,10,30,31,32,33,34,35,36,37,38]. Other studies have investigated the use of patterns to control the wetting of surfaces and membranes [30,40,41]. ElSherbiny et al. showed that 10–20 μm line-and-groove patterns connected by branches caused a slight decrease in concentration polarization on RO membranes [37]. In this study, we investigated the effect that nanosized surface patterns have on concentration polarization.

Recently, Zhou et al. performed computational studies on how micro- and nanopatterns affect concentration polarization on RO membranes [39]. They utilized different pattern shapes and sizes using computational fluid dynamics (CFD) to investigate how these patterns affected concentration polarization [39]. They found that the nanosized patterns did not impact concentration polarization, whereas, the micro-sized patterns elevated, not decreased, the concentration polarization effect compared to a perfectly flat membrane [39]. However, no membrane is perfectly flat, and therefore experiments need to be conducted to validate these models. Interestingly, Shang et al. recently reported using CFD models that predicted 100 μm triangular and cambered micro-patterned membranes would decrease concentration polarization [40]. These differing models provided motivation for this study to gain experimental results to compare to these computational studies.

Based on Zhou et al.’s results [39], we tested the hypothesis that nanosized line-and-groove patterns do not affect concentration polarization. A commercial NF membrane was patterned with a nanoscale line-and-groove silicon stamp by thermal embossing. The patterned membranes were characterized and tested to determine their pure water permeances and salt rejections at two salt concentrations. These values were compared with those of pristine NF membranes. Finally, the salt concentration at the membrane surface was estimated for both the pristine and nanopatterned membranes to determine whether nanosized patterns impact concentration polarization and to compare to the CFD results of Zhou et al. [39].

## 2. Materials and Methods

### 2.1. Materials

Polyamide thin-film composite NF270 membrane rolls were kindly provided by DuPont Water Solutions. NF270 consists of a polyester fabric backing, a polysulfone support layer, and a semi-aromatic polyamide selective layer [41]. The membrane was used as received. All membrane samples came from the center portion of the roll to avoid any edge defects that might be present. Sodium sulfate (Na_2_SO_4_, ≥99.0%, anhydrous) was purchased from VWR. Aqueous solutions were prepared with deionized water from a Millipore water purification system.

### 2.2. Membrane Patterning

Silicon line-and-groove stamps (29 mm × 12 mm) used to pattern the membranes were purchased from LightSmyth Technologies, Inc. The stamps were specified to have a 606 nm period between the peaks, a 190 nm groove depth, and a 303 nm line width [10,41]. See Weinman et al. for an image of the silicon stamp [10]. The membrane patterning procedure is similar to that of Weinman et al. with minor modifications [10,41]. Two stamps were placed side-by-side in contact with one another on top of the polyamide layer of the membrane. We placed the membrane and stamps on top of an 8 cm × 7.5 cm piece of 0.2 mm-thick aluminum shim (Grainger). A “cushion” of a 28.5 cm × 31 cm Kimwipe was folded to 1/16th its original size and placed on top of the membrane and stamps. This cushion was to help prevent the silicon stamps from breaking. Another similar size piece of the same aluminum shim was placed on top of the cushion and placed in a Carver press (Auto C-PL, HC 3889) to pattern the membrane. The press plates were heated to 45 °C and closed at a 25% pump speed until the pressure (force/stamp area) was 104 bar. The membrane was subject to this pressure for 15 min. The press did not hold a consistent pressure for the duration of the patterning process. The pressure slowly decreased to 90 bar before returning to the set pressure numerous times during the patterning time frame.

### 2.3. Membrane Characterization

#### 2.3.1. Atomic Force Microscopy

Atomic force microscopy (AFM) was utilized to observe the membrane surface before and after patterning. Images were taken using an Asylum Research MFP-3D AFM (Oxford Instruments) using MFP3D 14.48.159, Igor Pro 6.37 software. Pt-coated tip (radius 30 nm) cantilevers (NanoAndMore USA Corporation) were used for the non-contact tapping mode measurements. AFM images were taken with a 256 × 256 pixel resolution over 5 µm × 5 µm area at a scan rate of 1 Hz. The section analysis feature of the software was used to determine peak heights. The roughness analysis feature was used to determine membrane surface roughness.

#### 2.3.2. Scanning Electron Microscopy

Membrane top surface morphology before and after patterning was observed using a Thermo Fisher Apreo field-emission SEM (FE-SEM). Each membrane was attached to an aluminum stub with carbon tape and then gold coated prior to SEM measurements. The SEM measurements were performed at an accelerating voltage of 10.0 kV, a current voltage of 0.10 nA, and magnification of 12,000×.

### 2.4. Membrane Performance Testing

A stainless steel dead-end stirred cell from Sterlitech was used for membrane performance evaluation. The membrane testable area was 14.6 cm^2^. Before putting a membrane into the cell, the membrane was rinsed with DI water to remove any pore filler in the membrane. First, the membrane was tested with deionized water to determine the pure water permeance of the membrane. The solution temperature was 22–23 °C. The cell was pressurized by an air cylinder to 6.89 barg and permeate was allowed to flow for 30 min before sample collection to allow for membrane compaction. After the pure water test, the membrane was challenged with a 2000 ppm Na_2_SO_4_ solution. The same procedure was followed for the pure water permeance tests. This test was repeated at pressures of 6.89 barg, 10.34 barg, and 13.79 barg for a total of three testing pressures. After the 2000 ppm Na_2_SO_4_ tests, the same procedure was repeated with a 10,000 ppm Na_2_SO_4_ solution. All this testing was done on a single membrane. At least three pristine and three patterned membranes were tested to determine statistical significance. A conductivity meter (Traceable Conductivity Resistivity TDS Salinity Concentration Meter, VWR) was used to measure the feed and salt conductivities. A calibration curve was built as a function of known Na_2_SO_4_ concentration.

## 3. Theory

### NF Experiments

The standard flux and permeance model described by Equation (1) was used to calculate the permeance of each membrane for each experiment [42,43].

J_w_ = A(∆P−∆π) (1)

where J_w_ is the flux (L/m^2^/h or LMH) of the permeate solution, A is the membrane permeance (LMH/bar), ∆P is the difference in pressure (bar) between the feed and permeate (atmospheric pressure, 0 barg), and ∆π is the difference in osmotic pressure (bar) between the retentate and permeate. The flux of each membrane was calculated by dividing the permeate flow rate by the membrane testable area. For deionized water experiments, the flux was divided by the pressure difference (ΔP) to calculate the pure water permeance. For salt rejection experiments, the flux was divided by (ΔP−Δπ) to calculate the water permeance.

Since the salt concentration at the membrane surface cannot be directly measured in the direct flow cell, Equation (1) can be rearranged into Equation (2) to estimate the salt concentration located at the membrane surface.

(2) $$\pi_{m}=\Delta P-\frac{J_{w}}{A}+\pi_{p}$$

where π_m_ is the osmotic pressure (bar) at the membrane surface and π_P_ is the osmotic pressure (bar) of the permeate. We compared π_m_ between the pristine and patterned membranes. The osmotic pressure was then converted into Na_2_SO_4_ concentration using Equation (3), the Van’t Hoff equation, with i, the dissociation constant, equal to 3; R, the universal gas constant, equal to 0.08314 $\frac{L\times bar}{mol\times K}$; and T, temperature, equal to 22 °C or 295.15 K.

(3) $$C_{m}=\frac{\pi_{m}}{i\times R\times T}$$

The salt (Na_2_SO_4_) flux of the solution was calculated for each pressure and trial using Equation (4).

J_s_ = J_w_×C_p_ (4)

where J_s_ is the salt flux ($\frac{mol}{m^{2}*h}$), J_w_ is the water flux (LMH) that was calculated using Equation (1), and C_p_ is the Na_2_SO_4_ concentration of the permeate solution (mol/L).

## 4. Results and Discussion

### 4.1. Surface Patterning

**Figure 1:**
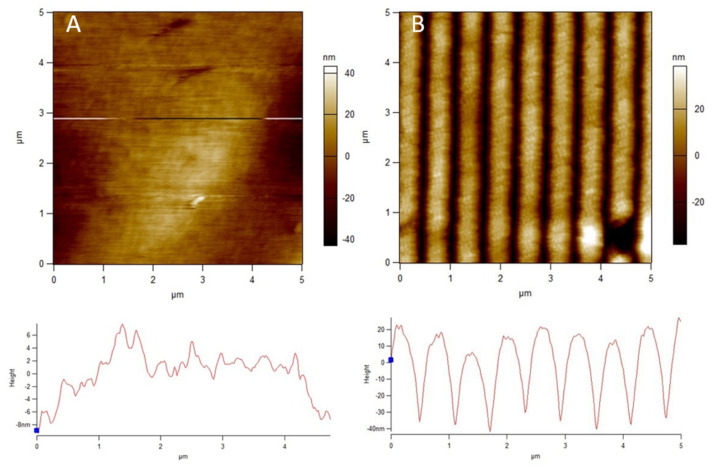
AFM images and cross-sectional profiles of a (**A**) pristine and (**B**) patterned NF270 membrane. The common scale is 5 μm × 5 μm.

**Figure 2:**
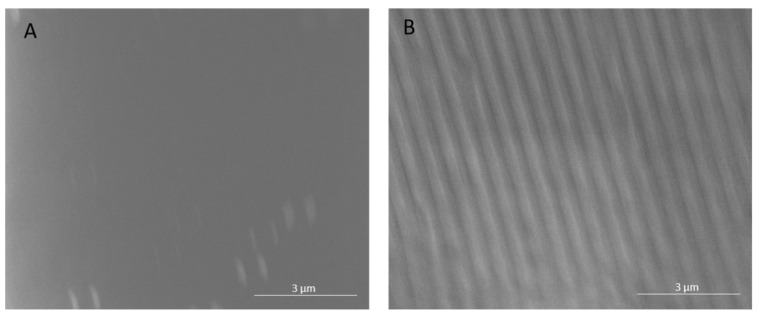
SEM images of a (**A**) pristine and (**B**) patterned NF270 membrane. Images were taken at 12,000× magnification and the common scale bar is 3 μm.

The polyamide NF270 membranes were directly patterned with silicon stamps by thermal embossing, which causes the active and support layers to deform into the pattern shape [10,11]. Therefore, the pattern on the membrane is the negative replica of the silicon stamp. Clear changes in the top surfaces of the membranes can be seen upon patterning in Figure 1 and Figure 2. Figure 1 shows AFM images of a pristine and patterned membrane, along with a cross-sectional profile of each image. As can be seen from the images, the membrane was clearly patterned in Figure 1B. The pattern peak height of the membrane was determined to be 55.5 ± 3.8 nm using the AFM sectional analysis tool. This average peak height is smaller than that found by Weinman et al. (144 nm) [41]; this is likely due to the use of a similar force over double the stamp size (therefore, roughly half the pressure). We did not increase the force of the press to achieve the same pressure as Weinman et al. due to concerns over breaking the stamps. Figure 2 shows the SEM images of the membranes. A clear, defined line-and-groove pattern is visible in the patterned membrane in Figure 2B.

### 4.2. Membrane Performance Properties

#### 4.2.1. Permeance and Salt Rejection

**Figure 3:**
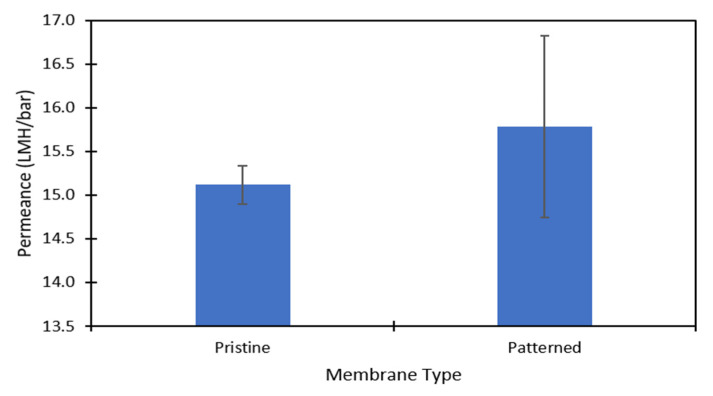
The pure water permeance (LMH/bar) data for the pristine and patterned membranes. The data was collected at 6.89 barg. The error bars represent one standard deviation among at least three membrane samples.

**Figure 4:**
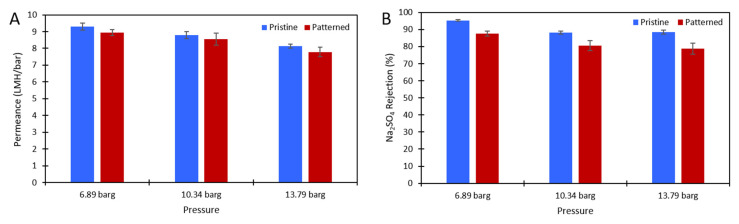
(**A**) The solution permeance (LMH/bar) data and (**B**) Na_2_SO_4_ rejection data for the 2000 ppm Na_2_SO_4_ feed. Blue is the pristine membrane, and red is the patterned membrane. The error bars represent one standard deviation among at least three membrane samples.

**Figure 5:**
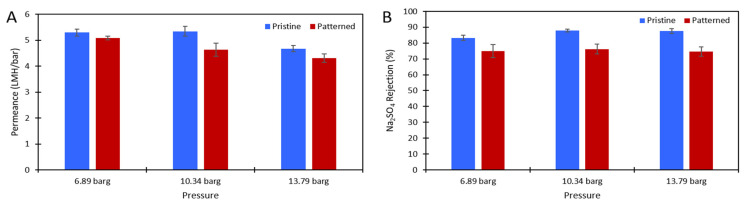
(**A**) The solution permeance (LMH/bar) data and (**B**) Na_2_SO_4_ rejection data for the 10,000 ppm Na_2_SO_4_ feed. Blue is the pristine membrane, and red is the patterned membrane. The error bars represent one standard deviation among at least three membrane samples.

DI water and aqueous solutions of Na_2_SO_4_ were used to challenge the membranes to ensure there was no damage to the polyamide layer after patterning and to test the effect of the nanopatterns on concentration polarization. Figure 3 shows the pure water permeance data of the pristine and patterned membranes that was collected at 6.89 barg. Figure 4 shows the permeance and rejection data of the pristine NF270 membranes and Figure 5 shows the permeance and rejection data of the patterned NF270 membranes. The data used to generate these figures are given in the supplementary materials. Not surprisingly, when the feed solution was changed from DI water to a salt solution, the permeance of both the pristine and patterned membranes decreased due to the osmotic pressure difference across the membrane from the salt. Similarly, when the feed concentration was increased, the rejection decreased. A paired two-sample *t*-test was done in Microsoft Excel to determine whether the permeance and rejection values were statistically different between the pristine and patterned membranes. Detailed results of these tests are given in Tables S6–S8 of the supplementary materials. The pure water permeances of the pristine and patterned membranes were not considered to be statistically different, indicating that the patterning process did not tear the polyamide active layer of the membrane. For both the 2000 ppm and 10,000 ppm Na_2_SO_4_ feed streams, it was determined that at any of the pressures tested, there was a statistically significantly lower rejection for the patterned membranes compared to the pristine membranes. This means that the patterning process was likely damaging the polyamide layer slightly, but not tearing it. This is similar to the findings of Weinman et al. when testing pristine and patterned NF270 membranes with MgSO_4_ [41]. Additionally, the patterned membrane permeance values were statistically significantly lower than the pristine membrane permeance values at 10.34 barg and 13.79 barg for the 10,000 ppm feed, indicating that the patterns did not decrease concentration polarization. The permeances of the pristine and patterned membranes for the 2000 ppm feed at all three testing pressures and the 10,000 ppm feed at 6.89 barg were not found to be statistically different.

**Figure 6:**
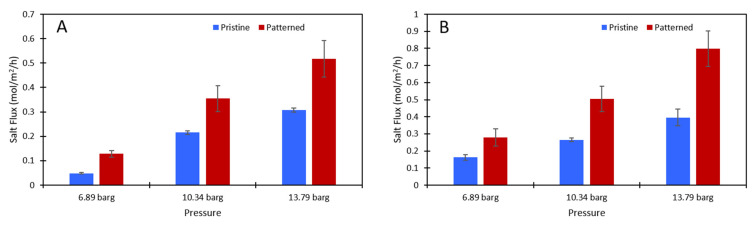
The salt flux (mol/m^2^/h) data for (**A**) the 2000 ppm Na_2_SO_4_ feed solution and (**B**) the 10,000 ppm Na_2_SO_4_ feed solution. Blue is the pristine membrane, and red is the patterned membrane. The error bars represent one standard deviation among at least three samples.

While the membranes were slightly damaged from the patterning, leading to a lower salt rejection, the patterned membranes had a statistically significantly higher salt flux (see Figure 6). The data used to generate this figure are given in the supplementary materials. Therefore, the amount of salt that could build up on the membrane was lower on the patterned membranes than on the pristine membranes because more salt was passing through the membrane. The impact of this on concentration polarization is discussed in the next section.

#### 4.2.2. Concentration Polarization

**Figure 7:**
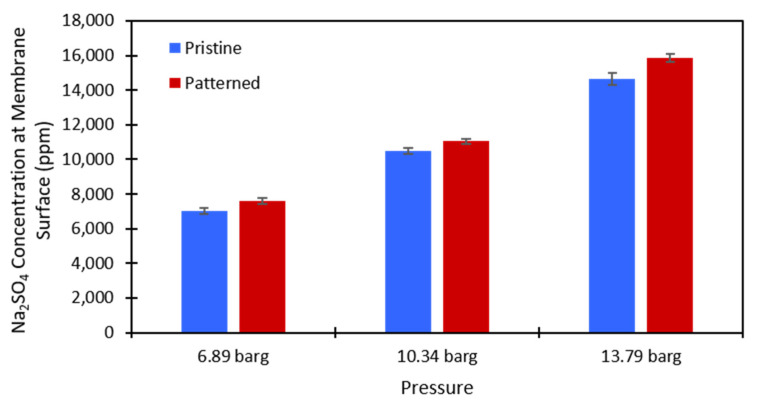
Calculated values of the Na_2_SO_4_ concentration at the membrane surface for the 2000 ppm feed stream. Blue is the pristine NF270 membrane, and red is the patterned NF270 membrane. The error bars represent one standard deviation among at least three membrane samples.

**Figure 8:**
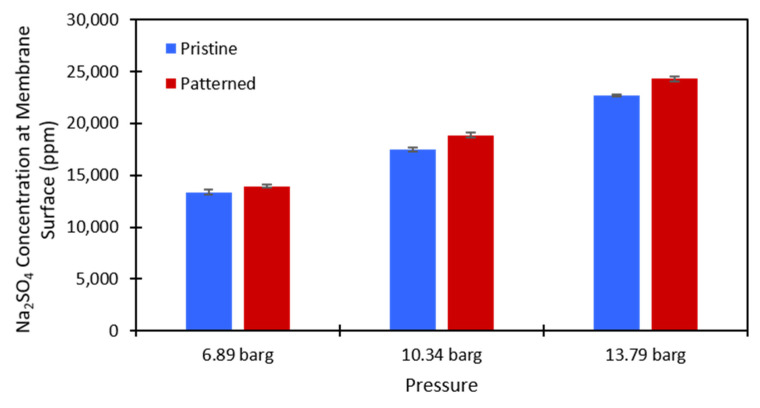
Calculated values of the Na_2_SO_4_ concentration at the membrane surface for the 10,000 ppm feed stream. Blue is the pristine NF270 membrane, and red is the patterned NF270 membrane. The error bars represent one standard deviation among at least three membrane samples.

Equation (3) was used to calculate the salt concentration at the membrane surface. There is no easy way to directly measure this value in a dead-end cell, so this method provided a simple way to estimate whether concentration polarization was affected by the presence of surface nanopatterns on the membrane. The calculations were performed at each testing pressure and each salt concentration that was used for both the pristine and patterned NF270 membranes. Figure 7 and Figure 8 show the calculated values for the 2000 ppm and 10,000 ppm Na_2_SO_4_ solutions, respectively. The data used to generate these figures are given in the supplementary materials. The Na_2_SO_4_ concentration at the membrane surface increased with the applied pressure, which is to be expected. As the pressure increases, more water molecules pass through the membrane, and more salt molecules are left behind to gather at/above the membrane. Additionally, the Na_2_SO_4_ concentration at the membrane surface increased with an increase in the feed concentration. This is expected because there is more salt that can be rejected by the membrane as the feed concentration is increased. The same paired two-sample *t*-test as described above was run on the salt concentration at the membrane surface for the pristine and patterned membranes. Detailed results of these tests are given in Tables S9 and S10 of the supplementary materials. It was found that for both feed concentrations at all tested pressures, the salt concentration at the membrane surface was statistically significantly higher for the patterned membranes compared to the pristine membranes. For the nanopatterns to positively impact concentration polarization, we needed to see a statistical decrease in the salt concentration at the membrane surface. This result indicates that the patterning does not help with concentration polarization, because even though the salt was passing through the patterned membranes at a higher rate, the estimated salt concentration at the membrane surface was higher for the patterned membranes. We suspect that if the salt fluxes were equal between the two membrane types, then the calculated concentration polarization (concentration at the membrane surface) would be even higher for the patterned membranes than what we measured. These results support the findings of Zhuo et al. that nano line-and-groove patterns do not positively impact the concentration polarization of salt-rejecting membranes [39], even though the pristine NF270 membranes are not perfectly flat as in their CFD simulations (we measured an RMS roughness value of 15.0 nm). The reasoning for these results is likely what Zhuo et al. state [39]. There is an increased salt concentration in the valleys of the line-and-groove nanopatterns and a lower salt concentration at the peaks of the line-and-groove patterns. Because the valleys take up more surface area than the peaks, the concentration polarization is worse for the patterned membranes than the pristine membranes. It is possible that an increased cross-flow rate across the pattern (stir speed in a dead-end cell or feed flow rate in a cross-flow cell) could induce the desired localized mixing. Additionally, the patterns might not be large enough to cause the desired localized mixing in the system. More work needs to be done to investigate these questions.

## 5. Conclusions

An established method was used to imprint a line-and-groove nanosized pattern onto a commercial NF membrane to test the hypothesis that nano-sized line-and-groove patterns do not affect concentration polarization. It was found that the nanopatterns did not decrease but increased the amount of concentration polarization at the membrane surface, thus disproving our hypothesis. This was the opposite of what was expected because it has been hypothesized that the increase in localized mixing due to the presence of the surface patterns would decrease the concentration polarization, as is seen in membrane fouling. This result could be due to there being a higher concentration located in the valleys of the patterns [39]. The patterning process did significantly decrease the salt rejection, which has been seen by other researchers. As seen with the increase in salt flux, patterning did not help decrease concentration polarization. Because of this higher salt flux, a lower amount of salt could build up at the patterned membrane surface, however, the calculated salt concentration at the membrane surface was higher for the patterned membranes than the pristine membranes. We suspect that if the salt fluxes were equal, then the concentration polarization would be even worse than what we found. More work is needed to investigate whether surface patterns on membranes can decrease concentration polarization due to the localized mixing effect. Ongoing work is investigating the effect of stir speed/cross-flow rate and the effect of micron-sized patterns and different pattern shapes on concentration polarization.

## Data Availability

The data presented in this study are available on request from the corresponding author.

## Supplementary Materials

The following are available online at https://www.mdpi.com/article/10.3390/membranes11120961/s1. Table S1: Permeance and Na_2_SO_4_ rejection values of the pristine NF270 membranes. Table S2. Permeance and Na_2_SO_4_ rejection values of the patterned NF270 membranes. Table S3. Salt flux values of the patterned and pristine NF270 membranes. Table S4. Concentration located at the membrane surface for the pristine NF270 membranes. Table S5. Concentration located at the membrane surface for the patterned NF270 membranes. Table S6. Results of paired two-sample *t*-test for the pure water feed solution. Table S7. Results of paired two-sample *t*-test for the 2000 ppm Na_2_SO_4_ feed solution. Table S8. Results of paired two-sample *t*-test for the 10,000 ppm Na_2_SO_4_ feed solution. Table S9. Results of paired two-sample *t*-test for the 2000 ppm Na_2_SO_4_ feed solution. Table S10. Results of paired two-sample *t*-test for the 10,000 ppm Na_2_SO_4_ feed solution.
