# Supplementary material for: Effect of Nanopatterning on Concentration Polarization during Nanofiltration
Source: Membranes (Basel). 2021 Dec 7;11(12):961. doi: 10.3390/membranes11120961 (PMC8707940; doi:10.3390/membranes11120961)
Supplement: Supplementary file 1 [file membranes-11-00961-s001.zip › membranes-1477811-supplementary.pdf]

Effect of Nanopatterning on Concentration Polarization during Nanofiltration

Lauren M. Ward [1], Barbara G. Fickling [1], and Steven T. Weinman [1,*]

Department of Chemical and Biological Engineering, The University of Alabama, Tuscaloosa, AL 35487, USA

Supporting Information

Permeance and Salt Rejection Data

The data in Table S1 and S2 were used to produce Figure 3, 4, and 5 in the main document.

Table S1. Permeance and Na_2SO_4 Rejection values of the pristine NF270 membranes. The error represents one standard deviation from a sample size of 3 for each test.

	Pristine Membrane				
	Pure Water Feed	2,000 ppm Feed		10,000 ppm Feed	
ΔP (bar)	Permeance (LMH/bar)	Permeance (LMH/bar)	Rejection (%)	Permeance (LMH/bar)	Rejection (%)
6.89	15.12 ± 0.05	9.29 ± 0.21	95.4 ± 0.4	5.29 ± 0.13	83.3 ± 1.6
10.34	-----	8.79 ± 0.22	88.3 ± 1.0	5.34 ± 0.18	87.9 ± 0.9
13.79	-----	8.13 ± 0.11	91.8 ± 4.7	4.68 ± 0.12	87.6 ± 1.6

Table S2. Permeance and Na_2SO_4 Rejection values of the patterned NF270 membranes. The error represents one standard deviation from a sample size of 3 for each test.

	Patterned Membrane				
	Pure Water Feed	2,000 ppm Feed		10,000 ppm Feed	
ΔP (bar)	Permeance (LMH/bar)	Permeance (LMH/bar)	Rejection (%)	Permeance (LMH/bar)	Rejection (%)
6.89	15.78 ± 1.09	8.95 ± 0.17	87.6 ± 1.4	5.08 ± 0.08	74.9 ± 4.1
10.34	-----	8.55 ± 0.36	81.0 ± 2.6	4.63 ± 0.25	76.1 ± 3.1
13.79	-----	7.78 ± 0.29	78.7 ± 3.2	4.31 ± 0.16	74.6 ± 2.8

Salt Flux Data

The data in Table S3 were used to produce Figure 6 in the main document.

Table S3. Salt flux data values for both pristine and patterned membranes. The error represents one standard deviation from a sample size of 3 for each test.

Salt Flux (mol/m²/hr)				
	2,000 ppm Feed		10,000 ppm Feed	
ΔP (bar)	Pristine	Patterned	Pristine	Patterned
6.89	0.05 ± 0.00	0.13 ± 0.01	0.16 ± 0.02	0.28 ± 0.05
10.34	0.22 ± 0.01	0.35 ± 0.05	0.27 ± 0.01	0.51 ± 0.07
13.79	0.31 ± 0.01	0.52 ± 0.07	0.40 ± 0.05	0.80 ± 0.10

Concentration Polarization Data

The data in Table S3 was used to produce Figure 5 in the main document. The data in Table S4 was used to produce Figure 6 in the main document.

Table S4. Concentration located at the membrane surface for the pristine NF270 membranes. The error represents one standard deviation from a sample size of 3 for each test.

Pristine Membrane		
	Concentration at the Membrane (ppm)	
ΔP (bar)	2,000 ppm Feed	10,000 ppm Feed
6.89	7029 ± 172	13379 ± 288
10.34	10493 ± 176	17475 ± 179
13.79	14639 ± 334	22723 ± 90

Table S5. Concentration located at the membrane surface for the patterned NF270 membranes. The error represents one standard deviation from a sample size of 3 for each test.

Patterned Membrane		
	Concentration at the Membrane (ppm)	
ΔP (bar)	2,000 ppm Feed	10,000 ppm Feed
6.89	7604 ± 153	13953 ± 110
10.34	11040 ± 155	18856 ± 244
13.79	15847 ± 236	24298 ± 265

Results from Paired Two sample t-test for Means

Hypothesis testing was done to determine statistical relevance of the data sets. EXCEL (Microsoft O365 Version 1908) was used for all statistical analyses. All tests were done using 90% confidence (α = 0.10); therefore, if the p-value is greater than α then the means are considered to be equal and if the p-value is less than α then the means are considered to be unequal.

Table S5 shows the results from the statistical tests on the pure water permeance data from Figures 3 and 4 in the main document (also Tables S1 and S2). Table S6 and S7 show the results from the statistical tests on the permeance and salt rejection data from Figure 3 in the main document (also Table S1) for the 2,000 ppm Na_2SO_4 feed and from Figure 4 in the main document (also Table S2) for the 10,000 ppm Na_2SO_4 feed. Table S8 and S9 show the results from the statistical tests on the calculated Na_2SO_4 concentration at the membrane surface from Figure 5 (also Table S3) for the 2,000 ppm Na_2SO_4 feed and Figure 6 (also Table S4) for the 10,000 ppm Na_2SO_4 feed in the main document.

Table S6. Results of paired two sample t-test for the pure water feed solution.

Pure Water Feed					
ΔP (bar)	Group 1 Data	Group 2 Data	Test Variable	Two-Tailed P Value	Interpretation (95% Confidence)
6.89	Pristine	Patterned	Permeance	0.354	Not statistically significant

Table S7. Results of paired two sample t-test for the 2,000 ppm Na_2SO_4 feed solution.

2,000 ppm Feed					
**ΔP (bar)**	**Group 1 Data**	**Group 2 Data**	**Test Variable**	**Two-Tailed P Value**	**Interpretation (95% Confidence)**
6.89	Pristine	Patterned	Permeance	0.244	Not statistically significant
10.34	Pristine	Patterned	Permeance	0.528	Not statistically significant
13.79	Pristine	Patterned	Permeance	0.143	Not statistically significant
6.89	Pristine	Patterned	Na_2SO_4 Rejection	0.011	Statistically significant
10.34	Pristine	Patterned	Na_2SO_4 Rejection	0.037	Statistically significant
13.79	Pristine	Patterned	Na_2SO_4 Rejection	0.020	Statistically significant
6.89	Pristine	Patterned	Na_2SO_4 Flux	0.016	Statistically significant
10.34	Pristine	Patterned	Na_2SO_4 Flux	0.058	Statistically significant
13.79	Pristine	Patterned	Na_2SO_4 Flux	0.053	Statistically significant

Table S8. Results of paired two sample t-test for the 10,000 ppm Na_2SO_4 feed solution.

10,000 ppm Feed					
**ΔP (bar)**	**Group 1 Data**	**Group 2 Data**	**Test Variable**	**Two-Tailed P Value**	**Interpretation (95% Confidence)**
6.89	Pristine	Patterned	Permeance	0.213	Not statistically significant
10.34	Pristine	Patterned	Permeance	0.099	Statistically significant
13.79	Pristine	Patterned	Permeance	0.032	Statistically significant
6.89	Pristine	Patterned	Na_2SO_4 Rejection	0.055	Statistically significant
10.34	Pristine	Patterned	Na_2SO_4 Rejection	0.025	Statistically significant
13.79	Pristine	Patterned	Na_2SO_4 Rejection	0.020	Statistically significant
6.89	Pristine	Patterned	Na_2SO_4 Flux	0.070	Statistically significant
10.34	Pristine	Patterned	Na_2SO_4 Flux	0.045	Statistically significant
13.79	Pristine	Patterned	Na_2SO_4 Flux	0.036	Statistically significant

Table S9. Results of paired two sample t-test for the 2,000 ppm Na_2SO_4 feed solution.

2,000 ppm Feed					
**ΔP (bar)**	**Group 1 Data**	**Group 2 Data**	**Test Variable**	**Two-Tailed P Value**	**Interpretation (95% Confidence)**
6.89	Pristine	Patterned	Na_2SO_4 Concentration at Membrane	0.088	Statistically significant
10.34	Pristine	Patterned	Na_2SO_4 Concentration at Membrane	0.094	Statistically significant
13.79	Pristine	Patterned	Na_2SO_4 Concentration at Membrane	0.002	Statistically significant

Table S10. Results of paired two sample t-test for the 10,000 ppm Na_2SO_4 feed solution.

10,000 ppm Feed					
**ΔP (bar)**	**Group 1 Data**	**Group 2 Data**	**Test Variable**	**Two-Tailed P Value**	**Interpretation (95% Confidence)**
6.89	Pristine	Patterned	Na_2SO_4 Concentration at Membrane	0.000	Statistically significant
10.34	Pristine	Patterned	Na_2SO_4 Concentration at Membrane	0.030	Statistically significant
13.79	Pristine	Patterned	Na_2SO_4 Concentration at Membrane	0.017	Statistically significant
